# Crystal structure of bis­(cyclo­hexyl­ammonium) di­phenyl­dioxalatostannate(IV)

**DOI:** 10.1107/S2056989014027716

**Published:** 2015-01-10

**Authors:** Modou Sarr, Aminata Diasse-Sarr, Libasse Diop, Laurent Plasseraud, Hélène Cattey

**Affiliations:** a Laboratoire de Chimie Minérale et Analytique (LACHIMIA), Département de Chimie, Faculté des Sciences et Techniques, Université Cheikh Anta Diop, Dakar, Senegal; b ICMUB UMR 6302, Université de Bourgogne, Faculté des Sciences, 9 avenue Alain Savary, 21000 Dijon, France

**Keywords:** crystal structure, organotin(IV) compound, oxalate ligands, *cis* arrangement, cyclo­hexyl­ammonium, N—H⋯O hydrogen bonding

## Abstract

Reaction of oxalic acid and di­phenyl­tin dichloride in the presence of cyclo­hexyl­amine led to the formation of the title salt, (C_6_H_14_N)_2_[Sn(C_6_H_5_)_2_(C_2_O_4_)_2_]. The dianion is made up from an Sn(C_6_H_5_)_2_ moiety *cis*-coordinated by two chelating oxalate anions, leading to an overall distorted octa­hedral coordination geometry of the Sn^IV^ atom. The negative charges are compensated by two surrounding cyclo­hexyl­ammonium cations adopting chair conformations each. In the crystal, anions and cations are linked *via* a network of N—H⋯O hydrogen bonds into a layered arrangement parallel to (101).

## Abstract 2

In the title salt, (CyNH_3_)_2_[Sn(Ph_2_)(C_2_O_4_)_2_] (Cy is cyclo­hexyl and Ph is phen­yl), the SnPh_2_ moiety is chelated by two oxalate anions, leading to a *cis* arrangement within the distorted octa­hedral coordination sphere of the Sn^IV^ atom.

## Chemical context   

Organotin(IV) complexes are particularly investigated for their catalytic applications as well as for their potential biocidal properties (Davies *et al.*, 2008 ▸). Thus, numerous studies have been carried out in order to determine the biological properties of organotin(IV) compounds against bacteria, fungi or cancer cell lines (Gielen, 2002 ▸). In this context, and in the course of our ongoing studies on organotin(IV) chemistry (Gueye *et al.*, 1993 ▸; Kane *et al.*, 2009 ▸; Fall, Okio *et al.*, 2010 ▸; Fall, Sow *et al.*, 2010 ▸), we have isolated the title stannate as colourless crystals from the reaction of oxalic acid and di­phenyl­tin dichloride in the presence of cyclo­hexyl­amine. To date, several organotin(IV) oxalates have been characterized by X-ray crystallographic analysis showing *cis*- and *trans*-coordination of the oxalate anion, depending on the nature of the σ-bonded carbon ligand that is linked to Sn^IV^ (Ng, 1996 ▸, 1999 ▸; Ng *et al.*, 1992 ▸; Ng & Hook, 1999 ▸; Ng & Rae, 2000 ▸; Xu *et al.*, 2003*a* ▸,*b* ▸; Gueye *et al.*, 2010 ▸, 2012 ▸; Reichelt & Reuter, 2014 ▸).
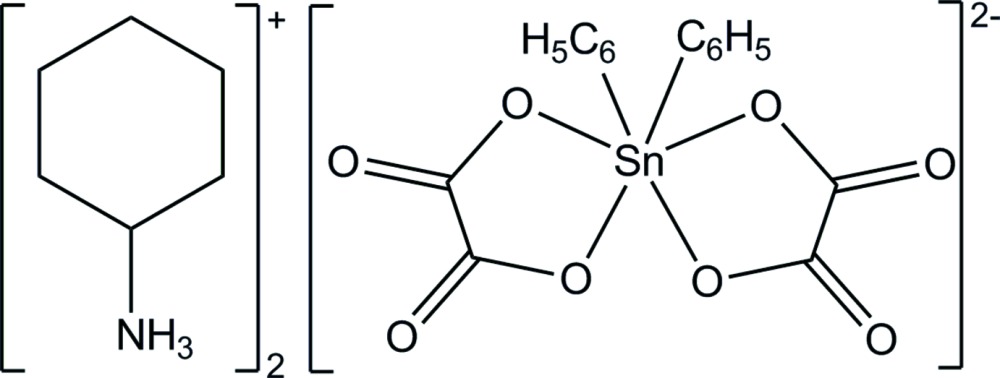


## Structural comment   

**Figure 1:**
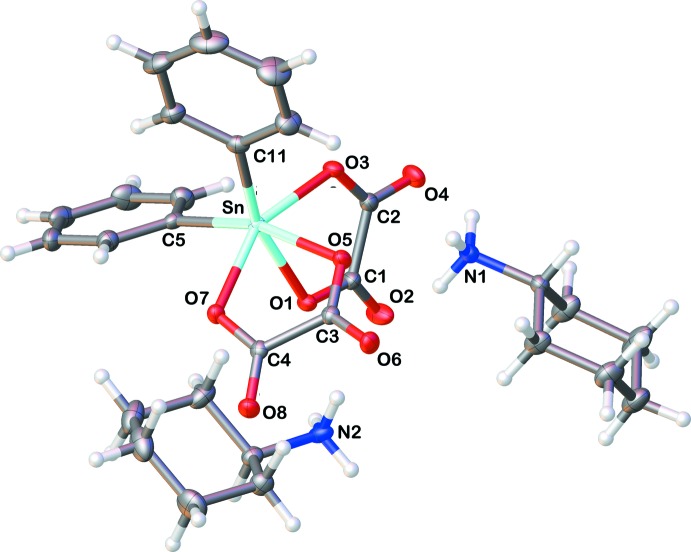
The mol­ecular components of the title salt, showing the atom labelling and with displacement ellipsoids drawn at the 30% probability level. Colour code: Sn = light blue, O = red, N = blue, C = grey and H = white.

In the title salt, 2(C_6_H_14_N)^+^[Sn(C_6_H_5_)_2_(C_2_O_4_)_2_]^2−^ or 2(CyNH_3_)^+^[Sn(Ph_2_)(C_2_O_4_)_2_]^2−^ (Cy is cyclo­hexyl; Ph is phen­yl), the SnPh_2_ moiety is chelated by two oxalate anions, leading to a *cis* arrangement within the distorted octa­hedral coordination sphere of the Sn^IV^ atom. The Sn—C distances and angles of the SnPh_2_ moiety [Sn—C5 = 2.1388 (15) Å, Sn—C11 = 2.1486 (15) Å with a C5—Sn—C11 angle of 106.94 (6)°] are similar to those previously reported for analogous di­phenyl­tin(IV) derivatives (Xu *et al.*, 2003*a* ▸,*b* ▸; Ng & Rae, 2000 ▸). The chelation of both oxalate anions is relatively symmetrical [Sn—O1 = 2.2005 (10) Å and Sn—O3 2.1267 (10) Å; Sn—O5 2.1883 (10) Å and Sn—O7 2.1396 (10) Å]. However, the oxalate anions are slightly distorted with O1—C1—C2—O3 and O5—C3—C4—O7 torsion angles of −4.0 (2) and −9.98 (19)°, respectively. They form a dihedral angle of 77.40 (8)° between their least-squares planes. The mol­ecular structure of the title compound, showing the atom-numbering scheme, is depicted in Fig. 1 ▸.

## Supra­molecular features   

**Figure 2:**
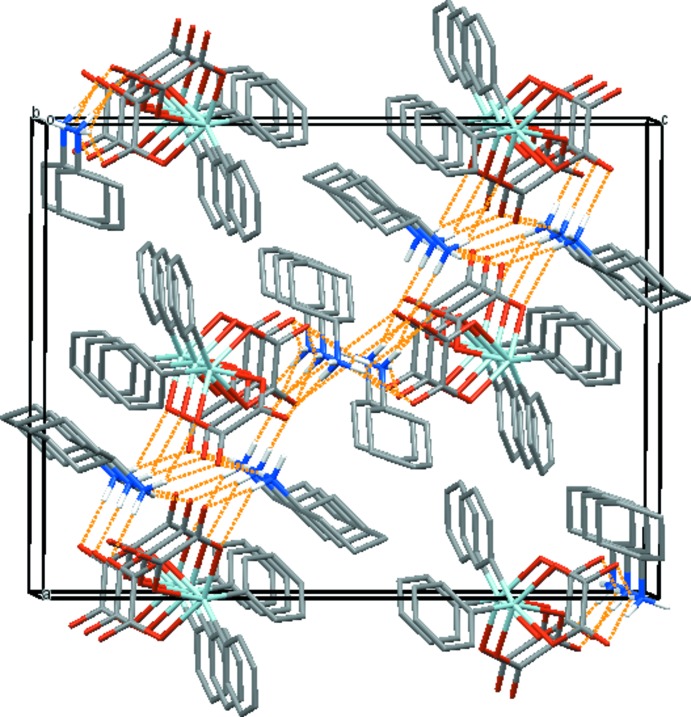
Crystal packing of the title compound, viewed approximately along the *b* axis, showing the layer-like arrangement parallel (101) *via* hydrogen-bonding inter­actions (dashed orange lines). H atoms not involved in hydrogen bonding have been omitted for clarity. Colour code: Sn = light blue, C = dark grey, H = white, N = dark blue and O = red.

From a supra­molecular point of view, anions and cations of the title salt exhibit inter­molecular inter­actions through N—H⋯O hydrogen-bonding contacts. Both coordinating and non-coordinating oxygen atoms of both oxalate anions are involved in these inter­actions (Table 1 ▸). Compared to the related structures of bis­(diiso­propyl­ammonium) [di­phenyl­dioxalatostannates(IV)] (Xu *et al.*, 2003*a* ▸,*b* ▸) where the supra­molecular arrangement defines infinite zigzag chains, the cyclo­hexyl­ammonium cations in the title structure lead to a layer-like arrangement parallel to (101) (Fig. 2 ▸).

## Synthesis and crystallization   

Chemicals were purchased from Sigma–Aldrich, and used without further purification. The title compound was obtained by reacting [(CyNH_3_)_2_C_2_O_4_]·1.5H_2_O – obtained previously in crystalline form by mixing CyNH_2_ with oxalic acid (C_2_O_4_H_2_) in a 2:1 molar ratio in water and evaporation at 333 K – with SnPh_2_Cl_2_ in methanol (molar ratio 2:1). Colourless single crystals suitable for X-ray diffraction analysis were obtained by slow solvent evaporation at room temperature.

## Refinement   

Crystal data, data collection and structure refinement details are summarized in Table 2 ▸. The H atoms bonded to C or N atoms were placed at calculated positions using a riding model with C—H = 0.95 (aromatic), 0.99 (methyl­ene) or N—H = 0.91 Å (amine) and with *U*_iso_(H) = 1.2*U*_eq_(C or N).

## Supplementary Material

Crystal structure: contains datablock(s) global, I. DOI: 10.1107/S2056989014027716/wm5103sup1.cif

Structure factors: contains datablock(s) I. DOI: 10.1107/S2056989014027716/wm5103Isup2.hkl

CCDC reference: 1040398

Additional supporting information:  crystallographic information; 3D view; checkCIF report

## Figures and Tables

**Table 1:** Hydrogen-bond geometry (, )

*D*H*A*	*D*H	H*A*	*D* *A*	*D*H*A*
N2H2*A*O2	0.91	1.90	2.7851(18)	163
N2H2*B*O6^i^	0.91	2.20	2.8885(17)	132
N2H2*B*O8^i^	0.91	2.23	3.0583(18)	151
N2H2*C*O6^ii^	0.91	2.68	3.2403(18)	121
N2H2*C*O8^ii^	0.91	2.01	2.8970(18)	164
N1H1*A*O6^i^	0.91	1.98	2.8842(17)	177
N1H1*B*O3^iii^	0.91	2.31	2.9393(16)	126
N1H1*B*O4^iii^	0.91	2.35	3.2550(18)	177
N1H1*C*O4	0.91	2.13	3.0076(18)	163

Symmetry codes: (i) 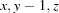; (ii) 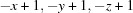; (iii) 
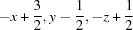
.

**Table 2:** Experimental details

Crystal data	
Chemical formula	(C_6_H_14_N)_2_[Sn(C_6_H_5_)_2_(C_2_O_2_)_2_]
*M*_r_	649.29
Crystal system, space group	Monoclinic, *P*2_1_/*n*
Temperature (K)	115
*a*, *b*, *c* ()	16.0084(6), 8.9010(3), 20.8060(8)
()	90.288(1)
*V* (^3^)	2964.63(19)
*Z*	4
Radiation type	Mo *K*
(mm^1^)	0.91
Crystal size (mm)	0.50 0.30 0.23

Data collection	
Diffractometer	Bruker APEXII CCD
Absorption correction	Multi-scan (*SADABS*; Bruker, 2014 ▸)
*T*_min_, *T*_max_	0.652, 0.746
No. of measured, independent and observed [*I* > 2(*I*)] reflections	30318, 6831, 6077
*R*_int_	0.025
(sin /)_max_ (^1^)	0.652

Refinement	
*R*[*F* ^2^ > 2(*F* ^2^)], *wR*(*F* ^2^), *S*	0.020, 0.048, 1.05
No. of reflections	6831
No. of parameters	354
H-atom treatment	H-atom parameters constrained
_max_, _min_ (e ^3^)	0.37, 0.38

Computer programs: *APEX2* and *SAINT* (Bruker, 2014 ▸), *SHELXS97* and *SHELXL97* (Sheldrick, 2008 ▸), *OLEX2* (Dolomanov *et al.*, 2009 ▸) and *Mercury* (Macrae *et al.*, 2008 ▸).

## supplementary crystallographic information

### Crystal data

**Table:**

(C_6_H_14_N)_2_[Sn(C_6_H_5_)_2_(C_2_O_2_)_2_]	*F*(000) = 1336
*M**_r_* = 649.29	*D*_x_ = 1.455 Mg m^−^^3^
Monoclinic, *P*2_1_/*n*	Mo *K*α radiation, λ = 0.71073 Å
Hall symbol: -P 2yn	Cell parameters from 9926 reflections
*a* = 16.0084 (6) Å	θ = 2.5–27.6°
*b* = 8.9010 (3) Å	µ = 0.91 mm^−^^1^
*c* = 20.8060 (8) Å	*T* = 115 K
β = 90.288 (1)°	Prism, colourless
*V* = 2964.63 (19) Å^3^	0.50 × 0.30 × 0.23 mm
*Z* = 4	

### Data collection

**Table:**

Bruker APEXII CCD diffractometer	6077 reflections with *I* > 2σ(*I*)
φ and ω scans	*R*_int_ = 0.025
Absorption correction: multi-scan (*SADABS*; Bruker, 2014)	θ_max_ = 27.6°, θ_min_ = 2.6°
*T*_min_ = 0.652, *T*_max_ = 0.746	*h* = −19→20
30318 measured reflections	*k* = −11→11
6831 independent reflections	*l* = −27→26

### Refinement

**Table:**

Refinement on *F*^2^	Primary atom site location: iterative
Least-squares matrix: full	Hydrogen site location: inferred from neighbouring sites
*R*[*F*^2^ > 2σ(*F*^2^)] = 0.020	H-atom parameters constrained
*wR*(*F*^2^) = 0.048	*w* = 1/[σ^2^(*F*_o_^2^) + (0.0188*P*)^2^ + 1.6859*P*] where *P* = (*F*_o_^2^ + 2*F*_c_^2^)/3
*S* = 1.05	(Δ/σ)_max_ = 0.003
6831 reflections	Δρ_max_ = 0.37 e Å^−^^3^
354 parameters	Δρ_min_ = −0.38 e Å^−^^3^
0 restraints	

### Special details

**Table:**

Geometry. All e.s.d.'s (except the e.s.d. in the dihedral angle between two l.s. planes) are estimated using the full covariance matrix. The cell e.s.d.'s are taken into account individually in the estimation of e.s.d.'s in distances, angles and torsion angles; correlations between e.s.d.'s in cell parameters are only used when they are defined by crystal symmetry. An approximate (isotropic) treatment of cell e.s.d.'s is used for estimating e.s.d.'s involving l.s. planes.

### Fractional atomic coordinates and isotropic or equivalent isotropic displacement parameters (Å^2^)

**Table:**

	*x*	*y*	*z*	*U*_iso_*/*U*_eq_	
Sn	0.51059 (2)	0.63627 (2)	0.26048 (2)	0.01224 (3)	
O1	0.53271 (6)	0.48705 (12)	0.34316 (5)	0.0161 (2)	
O2	0.60914 (8)	0.28994 (15)	0.37435 (6)	0.0298 (3)	
O3	0.62046 (6)	0.51474 (12)	0.23644 (5)	0.0167 (2)	
O4	0.70720 (7)	0.33267 (14)	0.26676 (6)	0.0253 (3)	
O5	0.59259 (6)	0.77093 (12)	0.32213 (5)	0.0168 (2)	
O6	0.58885 (7)	0.92654 (13)	0.40624 (5)	0.0194 (2)	
O7	0.42652 (6)	0.74042 (12)	0.32654 (5)	0.0154 (2)	
O8	0.42376 (7)	0.85828 (12)	0.42139 (5)	0.0198 (2)	
C1	0.59203 (9)	0.39309 (18)	0.33711 (8)	0.0178 (3)	
C2	0.64560 (9)	0.41207 (18)	0.27566 (7)	0.0171 (3)	
C3	0.55591 (9)	0.83851 (16)	0.36756 (7)	0.0140 (3)	
C4	0.46009 (9)	0.81040 (16)	0.37349 (7)	0.0136 (3)	
C5	0.41711 (9)	0.48308 (17)	0.22695 (7)	0.0152 (3)	
C6	0.43554 (11)	0.33624 (18)	0.20842 (8)	0.0228 (3)	
H6	0.4916	0.3013	0.2105	0.027*	
C7	0.37264 (12)	0.2402 (2)	0.18690 (9)	0.0296 (4)	
H7	0.3859	0.1402	0.1747	0.035*	
C8	0.29096 (11)	0.2901 (2)	0.18317 (8)	0.0281 (4)	
H8	0.2483	0.2247	0.1680	0.034*	
C9	0.27154 (10)	0.4346 (2)	0.20153 (8)	0.0256 (4)	
H9	0.2154	0.4688	0.1993	0.031*	
C10	0.33417 (10)	0.53031 (19)	0.22323 (8)	0.0200 (3)	
H10	0.3202	0.6298	0.2358	0.024*	
C11	0.52458 (10)	0.80526 (17)	0.18759 (8)	0.0169 (3)	
C12	0.46483 (11)	0.81749 (19)	0.13879 (8)	0.0229 (3)	
H12	0.4193	0.7491	0.1375	0.027*	
C13	0.47103 (13)	0.9286 (2)	0.09192 (9)	0.0319 (4)	
H13	0.4294	0.9365	0.0594	0.038*	
C14	0.53748 (14)	1.0273 (2)	0.09269 (10)	0.0371 (5)	
H14	0.5415	1.1035	0.0609	0.045*	
C15	0.59799 (13)	1.0150 (2)	0.13979 (10)	0.0375 (5)	
H15	0.6443	1.0817	0.1398	0.045*	
C16	0.59177 (11)	0.9056 (2)	0.18728 (9)	0.0268 (4)	
H16	0.6335	0.8991	0.2198	0.032*	
N2	0.48965 (9)	0.17026 (15)	0.45662 (6)	0.0198 (3)	
H2A	0.5239	0.2274	0.4318	0.024*	
H2B	0.4893	0.0742	0.4416	0.024*	
H2C	0.5085	0.1712	0.4979	0.024*	
C23	0.40312 (10)	0.23265 (17)	0.45409 (8)	0.0176 (3)	
H23	0.3656	0.1662	0.4798	0.021*	
C24	0.40273 (10)	0.38860 (18)	0.48363 (8)	0.0226 (3)	
H24A	0.4429	0.4536	0.4607	0.027*	
H24B	0.4204	0.3824	0.5292	0.027*	
C25	0.31532 (12)	0.4573 (2)	0.47938 (10)	0.0357 (5)	
H25A	0.2763	0.3981	0.5061	0.043*	
H25B	0.3167	0.5612	0.4963	0.043*	
C26	0.28439 (11)	0.4592 (2)	0.41009 (11)	0.0357 (5)	
H26A	0.2268	0.4995	0.4087	0.043*	
H26B	0.3204	0.5265	0.3844	0.043*	
C27	0.28533 (12)	0.3030 (2)	0.38087 (10)	0.0345 (5)	
H27A	0.2675	0.3087	0.3353	0.041*	
H27B	0.2453	0.2380	0.4040	0.041*	
C28	0.37231 (11)	0.23443 (19)	0.38491 (8)	0.0257 (4)	
H28A	0.3708	0.1305	0.3680	0.031*	
H28B	0.4113	0.2936	0.3582	0.031*	
N1	0.73487 (8)	0.05755 (15)	0.34805 (6)	0.0182 (3)	
H1A	0.6877	0.0187	0.3657	0.022*	
H1B	0.7531	−0.0044	0.3163	0.022*	
H1C	0.7236	0.1499	0.3313	0.022*	
C17	0.80130 (9)	0.07153 (18)	0.39886 (7)	0.0171 (3)	
H17	0.8533	0.1107	0.3783	0.021*	
C18	0.81959 (12)	−0.0828 (2)	0.42605 (9)	0.0288 (4)	
H18A	0.8401	−0.1493	0.3914	0.035*	
H18B	0.7676	−0.1269	0.4434	0.035*	
C19	0.88557 (14)	−0.0728 (2)	0.47965 (10)	0.0412 (5)	
H19A	0.8935	−0.1733	0.4991	0.049*	
H19B	0.9395	−0.0409	0.4610	0.049*	
C20	0.85982 (12)	0.0381 (2)	0.53148 (9)	0.0319 (4)	
H20A	0.8093	0.0005	0.5535	0.038*	
H20B	0.9051	0.0470	0.5638	0.038*	
C21	0.84186 (12)	0.1913 (2)	0.50270 (9)	0.0309 (4)	
H21A	0.8937	0.2327	0.4840	0.037*	
H21B	0.8231	0.2604	0.5370	0.037*	
C22	0.77453 (11)	0.18142 (19)	0.45050 (8)	0.0241 (4)	
H22A	0.7213	0.1477	0.4697	0.029*	
H22B	0.7655	0.2818	0.4312	0.029*	

### Atomic displacement parameters (Å^2^)

**Table:**

	*U*^11^	*U*^22^	*U*^33^	*U*^12^	*U*^13^	*U*^23^
Sn	0.01278 (5)	0.01303 (5)	0.01090 (5)	−0.00039 (4)	0.00020 (3)	−0.00061 (4)
O1	0.0153 (5)	0.0196 (5)	0.0135 (5)	0.0037 (4)	0.0023 (4)	0.0018 (4)
O2	0.0287 (6)	0.0363 (7)	0.0244 (7)	0.0157 (5)	0.0075 (5)	0.0145 (6)
O3	0.0166 (5)	0.0200 (5)	0.0136 (5)	0.0018 (4)	0.0040 (4)	0.0023 (4)
O4	0.0175 (6)	0.0329 (7)	0.0256 (7)	0.0097 (5)	0.0053 (5)	0.0033 (5)
O5	0.0138 (5)	0.0205 (6)	0.0161 (6)	−0.0015 (4)	0.0009 (4)	−0.0040 (4)
O6	0.0173 (5)	0.0221 (6)	0.0189 (6)	−0.0023 (4)	−0.0023 (4)	−0.0056 (5)
O7	0.0132 (5)	0.0170 (5)	0.0160 (5)	0.0004 (4)	−0.0002 (4)	−0.0035 (4)
O8	0.0180 (5)	0.0244 (6)	0.0170 (6)	0.0019 (4)	0.0022 (4)	−0.0050 (5)
C1	0.0152 (7)	0.0237 (8)	0.0145 (7)	0.0021 (6)	−0.0001 (6)	0.0022 (6)
C2	0.0140 (7)	0.0216 (8)	0.0157 (7)	−0.0006 (6)	0.0005 (6)	−0.0009 (6)
C3	0.0153 (7)	0.0124 (7)	0.0141 (7)	0.0005 (5)	−0.0010 (6)	0.0022 (6)
C4	0.0146 (7)	0.0113 (6)	0.0149 (7)	0.0014 (5)	−0.0010 (6)	0.0019 (6)
C5	0.0189 (7)	0.0160 (7)	0.0107 (7)	−0.0021 (6)	−0.0001 (6)	0.0001 (6)
C6	0.0234 (8)	0.0214 (8)	0.0237 (9)	0.0003 (6)	0.0018 (7)	−0.0039 (7)
C7	0.0395 (10)	0.0205 (8)	0.0289 (10)	−0.0076 (7)	0.0048 (8)	−0.0095 (7)
C8	0.0300 (9)	0.0341 (10)	0.0201 (9)	−0.0173 (8)	0.0009 (7)	−0.0047 (7)
C9	0.0185 (8)	0.0368 (10)	0.0214 (9)	−0.0059 (7)	−0.0022 (6)	0.0000 (8)
C10	0.0212 (8)	0.0197 (8)	0.0191 (8)	−0.0007 (6)	−0.0005 (6)	−0.0009 (6)
C11	0.0212 (8)	0.0139 (7)	0.0157 (8)	0.0027 (6)	0.0044 (6)	−0.0002 (6)
C12	0.0270 (9)	0.0234 (8)	0.0182 (8)	0.0011 (7)	0.0006 (7)	0.0012 (7)
C13	0.0446 (11)	0.0328 (10)	0.0183 (9)	0.0123 (9)	0.0001 (8)	0.0063 (8)
C14	0.0603 (13)	0.0227 (9)	0.0285 (10)	0.0055 (9)	0.0130 (9)	0.0110 (8)
C15	0.0461 (12)	0.0260 (10)	0.0404 (12)	−0.0105 (9)	0.0076 (9)	0.0091 (9)
C16	0.0290 (9)	0.0237 (8)	0.0276 (9)	−0.0059 (7)	0.0011 (7)	0.0037 (7)
N2	0.0283 (7)	0.0188 (7)	0.0122 (6)	0.0068 (5)	0.0016 (5)	0.0004 (5)
C23	0.0204 (8)	0.0162 (7)	0.0163 (8)	−0.0004 (6)	0.0000 (6)	0.0020 (6)
C24	0.0250 (8)	0.0205 (8)	0.0223 (8)	0.0034 (6)	0.0003 (7)	−0.0032 (7)
C25	0.0312 (10)	0.0303 (10)	0.0456 (12)	0.0117 (8)	0.0072 (9)	−0.0008 (9)
C26	0.0232 (9)	0.0321 (10)	0.0516 (13)	0.0037 (8)	−0.0037 (9)	0.0154 (9)
C27	0.0308 (10)	0.0331 (10)	0.0394 (12)	−0.0095 (8)	−0.0151 (8)	0.0140 (9)
C28	0.0357 (10)	0.0220 (8)	0.0192 (8)	−0.0032 (7)	−0.0070 (7)	0.0023 (7)
N1	0.0158 (6)	0.0220 (7)	0.0167 (7)	0.0046 (5)	0.0022 (5)	0.0002 (5)
C17	0.0149 (7)	0.0204 (8)	0.0160 (8)	0.0006 (6)	0.0012 (6)	0.0006 (6)
C18	0.0408 (10)	0.0214 (8)	0.0243 (9)	0.0120 (8)	−0.0058 (8)	−0.0023 (7)
C19	0.0523 (13)	0.0412 (12)	0.0299 (11)	0.0266 (10)	−0.0150 (9)	−0.0054 (9)
C20	0.0395 (11)	0.0367 (10)	0.0195 (9)	0.0080 (8)	−0.0060 (8)	−0.0013 (8)
C21	0.0413 (11)	0.0280 (9)	0.0234 (9)	−0.0007 (8)	−0.0047 (8)	−0.0050 (8)
C22	0.0314 (9)	0.0192 (8)	0.0216 (9)	0.0076 (7)	−0.0003 (7)	−0.0029 (7)

### Geometric parameters (Å, º)

**Table:**

Sn—O1	2.2005 (10)	N2—H2C	0.9100
Sn—O3	2.1267 (10)	N2—C23	1.493 (2)
Sn—O5	2.1883 (10)	C23—H23	1.0000
Sn—O7	2.1396 (10)	C23—C24	1.518 (2)
Sn—C5	2.1388 (15)	C23—C28	1.519 (2)
Sn—C11	2.1486 (15)	C24—H24A	0.9900
O1—C1	1.2721 (18)	C24—H24B	0.9900
O2—C1	1.2312 (19)	C24—C25	1.529 (2)
O3—C2	1.2883 (19)	C25—H25A	0.9900
O4—C2	1.2280 (19)	C25—H25B	0.9900
O5—C3	1.2672 (18)	C25—C26	1.522 (3)
O6—C3	1.2391 (18)	C26—H26A	0.9900
O7—C4	1.2751 (18)	C26—H26B	0.9900
O8—C4	1.2326 (18)	C26—C27	1.517 (3)
C1—C2	1.552 (2)	C27—H27A	0.9900
C3—C4	1.560 (2)	C27—H27B	0.9900
C5—C6	1.395 (2)	C27—C28	1.522 (3)
C5—C10	1.394 (2)	C28—H28A	0.9900
C6—H6	0.9500	C28—H28B	0.9900
C6—C7	1.393 (2)	N1—H1A	0.9100
C7—H7	0.9500	N1—H1B	0.9100
C7—C8	1.383 (3)	N1—H1C	0.9100
C8—H8	0.9500	N1—C17	1.5009 (19)
C8—C9	1.378 (3)	C17—H17	1.0000
C9—H9	0.9500	C17—C18	1.514 (2)
C9—C10	1.389 (2)	C17—C22	1.516 (2)
C10—H10	0.9500	C18—H18A	0.9900
C11—C12	1.396 (2)	C18—H18B	0.9900
C11—C16	1.398 (2)	C18—C19	1.535 (3)
C12—H12	0.9500	C19—H19A	0.9900
C12—C13	1.393 (2)	C19—H19B	0.9900
C13—H13	0.9500	C19—C20	1.521 (3)
C13—C14	1.380 (3)	C20—H20A	0.9900
C14—H14	0.9500	C20—H20B	0.9900
C14—C15	1.379 (3)	C20—C21	1.516 (3)
C15—H15	0.9500	C21—H21A	0.9900
C15—C16	1.391 (3)	C21—H21B	0.9900
C16—H16	0.9500	C21—C22	1.529 (2)
N2—H2A	0.9100	C22—H22A	0.9900
N2—H2B	0.9100	C22—H22B	0.9900
			
O3—Sn—O1	75.33 (4)	C24—C23—H23	108.7
O3—Sn—O5	85.53 (4)	C24—C23—C28	111.84 (13)
O3—Sn—O7	153.52 (4)	C28—C23—H23	108.7
O3—Sn—C5	100.20 (5)	C23—C24—H24A	109.6
O3—Sn—C11	95.78 (5)	C23—C24—H24B	109.6
O5—Sn—O1	77.21 (4)	C23—C24—C25	110.36 (14)
O7—Sn—O1	81.88 (4)	H24A—C24—H24B	108.1
O7—Sn—O5	76.33 (4)	C25—C24—H24A	109.6
O7—Sn—C11	102.60 (5)	C25—C24—H24B	109.6
C5—Sn—O1	88.84 (5)	C24—C25—H25A	109.5
C5—Sn—O5	163.15 (5)	C24—C25—H25B	109.5
C5—Sn—O7	92.55 (5)	H25A—C25—H25B	108.1
C5—Sn—C11	106.94 (6)	C26—C25—C24	110.63 (16)
C11—Sn—O1	163.22 (5)	C26—C25—H25A	109.5
C11—Sn—O5	88.05 (5)	C26—C25—H25B	109.5
C1—O1—Sn	115.90 (9)	C25—C26—H26A	109.3
C2—O3—Sn	117.90 (9)	C25—C26—H26B	109.3
C3—O5—Sn	114.73 (9)	H26A—C26—H26B	108.0
C4—O7—Sn	116.08 (9)	C27—C26—C25	111.46 (16)
O1—C1—C2	115.19 (13)	C27—C26—H26A	109.3
O2—C1—O1	126.29 (15)	C27—C26—H26B	109.3
O2—C1—C2	118.51 (14)	C26—C27—H27A	109.5
O3—C2—C1	115.26 (13)	C26—C27—H27B	109.5
O4—C2—O3	124.14 (14)	C26—C27—C28	110.87 (15)
O4—C2—C1	120.59 (14)	H27A—C27—H27B	108.1
O5—C3—C4	116.26 (13)	C28—C27—H27A	109.5
O6—C3—O5	125.96 (14)	C28—C27—H27B	109.5
O6—C3—C4	117.75 (13)	C23—C28—C27	110.43 (15)
O7—C4—C3	115.39 (13)	C23—C28—H28A	109.6
O8—C4—O7	126.13 (14)	C23—C28—H28B	109.6
O8—C4—C3	118.47 (13)	C27—C28—H28A	109.6
C6—C5—Sn	122.61 (12)	C27—C28—H28B	109.6
C10—C5—Sn	119.36 (11)	H28A—C28—H28B	108.1
C10—C5—C6	118.02 (14)	H1A—N1—H1B	109.5
C5—C6—H6	119.7	H1A—N1—H1C	109.5
C7—C6—C5	120.68 (16)	H1B—N1—H1C	109.5
C7—C6—H6	119.7	C17—N1—H1A	109.5
C6—C7—H7	119.9	C17—N1—H1B	109.5
C8—C7—C6	120.20 (16)	C17—N1—H1C	109.5
C8—C7—H7	119.9	N1—C17—H17	108.4
C7—C8—H8	120.0	N1—C17—C18	108.84 (13)
C9—C8—C7	119.91 (16)	N1—C17—C22	110.54 (12)
C9—C8—H8	120.0	C18—C17—H17	108.4
C8—C9—H9	120.0	C18—C17—C22	112.06 (14)
C8—C9—C10	119.93 (16)	C22—C17—H17	108.4
C10—C9—H9	120.0	C17—C18—H18A	109.6
C5—C10—H10	119.4	C17—C18—H18B	109.6
C9—C10—C5	121.24 (15)	C17—C18—C19	110.48 (16)
C9—C10—H10	119.4	H18A—C18—H18B	108.1
C12—C11—Sn	119.65 (11)	C19—C18—H18A	109.6
C12—C11—C16	118.11 (15)	C19—C18—H18B	109.6
C16—C11—Sn	122.24 (12)	C18—C19—H19A	109.3
C11—C12—H12	119.5	C18—C19—H19B	109.3
C13—C12—C11	120.91 (17)	H19A—C19—H19B	108.0
C13—C12—H12	119.5	C20—C19—C18	111.41 (15)
C12—C13—H13	119.9	C20—C19—H19A	109.3
C14—C13—C12	120.11 (18)	C20—C19—H19B	109.3
C14—C13—H13	119.9	C19—C20—H20A	109.5
C13—C14—H14	120.1	C19—C20—H20B	109.5
C15—C14—C13	119.77 (17)	H20A—C20—H20B	108.1
C15—C14—H14	120.1	C21—C20—C19	110.81 (16)
C14—C15—H15	119.7	C21—C20—H20A	109.5
C14—C15—C16	120.53 (18)	C21—C20—H20B	109.5
C16—C15—H15	119.7	C20—C21—H21A	109.4
C11—C16—H16	119.7	C20—C21—H21B	109.4
C15—C16—C11	120.54 (18)	C20—C21—C22	111.15 (15)
C15—C16—H16	119.7	H21A—C21—H21B	108.0
H2A—N2—H2B	109.5	C22—C21—H21A	109.4
H2A—N2—H2C	109.5	C22—C21—H21B	109.4
H2B—N2—H2C	109.5	C17—C22—C21	109.86 (14)
C23—N2—H2A	109.5	C17—C22—H22A	109.7
C23—N2—H2B	109.5	C17—C22—H22B	109.7
C23—N2—H2C	109.5	C21—C22—H22A	109.7
N2—C23—H23	108.7	C21—C22—H22B	109.7
N2—C23—C24	109.37 (13)	H22A—C22—H22B	108.2
N2—C23—C28	109.51 (13)		
			
Sn—O1—C1—O2	−171.88 (14)	C8—C9—C10—C5	0.0 (3)
Sn—O1—C1—C2	6.94 (17)	C10—C5—C6—C7	0.0 (2)
Sn—O3—C2—O4	178.78 (12)	C11—C12—C13—C14	0.9 (3)
Sn—O3—C2—C1	−1.22 (17)	C12—C11—C16—C15	0.5 (3)
Sn—O5—C3—O6	−176.13 (12)	C12—C13—C14—C15	0.4 (3)
Sn—O5—C3—C4	2.10 (16)	C13—C14—C15—C16	−1.2 (3)
Sn—O7—C4—O8	−168.51 (12)	C14—C15—C16—C11	0.8 (3)
Sn—O7—C4—C3	12.63 (16)	C16—C11—C12—C13	−1.4 (2)
Sn—C5—C6—C7	179.96 (13)	N2—C23—C24—C25	177.93 (14)
Sn—C5—C10—C9	179.79 (12)	N2—C23—C28—C27	−177.92 (13)
Sn—C11—C12—C13	178.20 (13)	C23—C24—C25—C26	−55.7 (2)
Sn—C11—C16—C15	−179.04 (14)	C24—C23—C28—C27	−56.52 (18)
O1—C1—C2—O3	−4.0 (2)	C24—C25—C26—C27	56.3 (2)
O1—C1—C2—O4	176.02 (15)	C25—C26—C27—C28	−56.5 (2)
O2—C1—C2—O3	174.93 (15)	C26—C27—C28—C23	55.9 (2)
O2—C1—C2—O4	−5.1 (2)	C28—C23—C24—C25	56.44 (19)
O5—C3—C4—O7	−9.98 (19)	N1—C17—C18—C19	−178.38 (14)
O5—C3—C4—O8	171.06 (13)	N1—C17—C22—C21	178.56 (14)
O6—C3—C4—O7	168.40 (13)	C17—C18—C19—C20	54.6 (2)
O6—C3—C4—O8	−10.6 (2)	C18—C17—C22—C21	56.96 (19)
C5—C6—C7—C8	0.5 (3)	C18—C19—C20—C21	−55.5 (2)
C6—C5—C10—C9	−0.2 (2)	C19—C20—C21—C22	56.9 (2)
C6—C7—C8—C9	−0.8 (3)	C20—C21—C22—C17	−57.1 (2)
C7—C8—C9—C10	0.5 (3)	C22—C17—C18—C19	−55.8 (2)

### Hydrogen-bond geometry (Å, º)

**Table:**

*D*—H···*A*	*D*—H	H···*A*	*D*···*A*	*D*—H···*A*
N2—H2*A*···O2	0.91	1.90	2.7851 (18)	163
N2—H2*B*···O6^i^	0.91	2.20	2.8885 (17)	132
N2—H2*B*···O8^i^	0.91	2.23	3.0583 (18)	151
N2—H2*C*···O6^ii^	0.91	2.68	3.2403 (18)	121
N2—H2*C*···O8^ii^	0.91	2.01	2.8970 (18)	164
N1—H1*A*···O6^i^	0.91	1.98	2.8842 (17)	177
N1—H1*B*···O3^iii^	0.91	2.31	2.9393 (16)	126
N1—H1*B*···O4^iii^	0.91	2.35	3.2550 (18)	177
N1—H1*C*···O4	0.91	2.13	3.0076 (18)	163

Symmetry codes: (i) *x*, *y*−1, *z*; (ii) −*x*+1, −*y*+1, −*z*+1; (iii) −*x*+3/2, *y*−1/2, −*z*+1/2.
